# Promoter methylation correlates with reduced *NDRG2 *expression in advanced colon tumour

**DOI:** 10.1186/1755-8794-2-11

**Published:** 2009-03-03

**Authors:** Ada Piepoli, Rosa Cotugno, Giuseppe Merla, Annamaria Gentile, Bartolomeo Augello, Michele Quitadamo, Antonio Merla, Anna Panza, Massimo Carella, Rosalia Maglietta, Annarita D'Addabbo, Nicola Ancona, Saverio Fusilli, Francesco Perri, Angelo Andriulli

**Affiliations:** 1 Gastroenterology Unit and Research Laboratory, "Casa Sollievo della Sofferenza", Hospital, IRCCS, San Giovanni Rotondo, Italy; 2 Medical Genetics Service, "Casa Sollievo della Sofferenza", Hospital, IRCCS, San Giovanni Rotondo, Italy; 3 Istituto di Studi sui Sistemi Intelligenti per l'Automazione – CNR, Bari, Italy; 4 Health Services, "Casa Sollievo della Sofferenza", Hospital, IRCCS, San Giovanni Rotondo, Italy

## Abstract

**Background:**

Aberrant DNA methylation of CpG islands of cancer-related genes is among the earliest and most frequent alterations in cancerogenesis and might be of value for either diagnosing cancer or evaluating recurrent disease. This mechanism usually leads to inactivation of tumour-suppressor genes. We have designed the current study to validate our previous microarray data and to identify novel hypermethylated gene promoters.

**Methods:**

The validation assay was performed in a different set of 8 patients with colorectal cancer (CRC) by means quantitative reverse-transcriptase polymerase chain reaction analysis. The differential RNA expression profiles of three CRC cell lines before and after 5-aza-2'-deoxycytidine treatment were compared to identify the hypermethylated genes. The DNA methylation status of these genes was evaluated by means of bisulphite genomic sequencing and methylation-specific polymerase chain reaction (MSP) in the 3 cell lines and in tumour tissues from 30 patients with CRC.

**Results:**

Data from our previous genome search have received confirmation in the new set of 8 patients with CRC. In this validation set six genes showed a high induction after drug treatment in at least two of three CRC cell lines. Among them, the N-myc downstream-regulated gene 2 (*NDRG2) *promoter was found methylated in all CRC cell lines. *NDRG2 *hypermethylation was also detected in 8 out of 30 (27%) primary CRC tissues and was significantly associated with advanced AJCC stage IV. Normal colon tissues were not methylated.

**Conclusion:**

The findings highlight the usefulness of combining gene expression patterns and epigenetic data to identify tumour biomarkers, and suggest that NDRG2 silencing might bear influence on tumour invasiveness, being associated with a more advanced stage.

## Background

Colorectal cancer (CRC) is the third most common cancer in men and women, accounting for 11% of all cancer-related deaths. The majority of cases are diagnosed in advanced stages when a curative treatment is less likely to occur and chemotherapy is the only option [1]. The identification of the molecular, genetic, and epigenetic changes underlying the adenoma-carcinoma sequence [2] leading to CRC has been the focus of many researches [3]. It is now widely accepted that sporadic CRC frequently arises from preneoplastic lesions through the activation of proto-oncogenes, such as K-ras, and the inactivation of tumor suppressor genes (TSG), such as APC, p53, DCC, and the mismatch repair genes [4,5]. Apart from mutations, gene expression may also be modified by altering of DNA methylation [6]. Two general phenomena have until now been observed. The first one is global DNA hypomethylation with decreased 5-methylcytosine content which results in both enhanced expression of proto-oncogenes [7] and genomic instability [8]. The second event is represented by local DNA hypermethylation of CpG islands, short sequences rich in CpG dinucleotides in the 5'untranscribed region (5'-UTR). This event occurs in approximately half of all human genes [9,10] silences specific TSG, and accelerates cancer formation [11,12]. Treatment with DNA demethylating drugs, such as 5-aza-2'-deoxycytidine (5-Aza-CdR or Decitabine), was shown to reverse the hypermethylation and restore expression of TSG [13]. Therefore, cancer-specific promoter methylation may by itself serve as a valuable clue to uncover novel TSG.

In the present study, we aimed to uncover novel targets of promoter methylation in CRC, by combining gene expression profile data, already highlighted by our group [14], with results of demethylating assay and *in silico *screening for CpG islands.

## Methods

### Patients

Peripheral blood, primary tumour and matching normal tissue samples from a cohort of 30 consecutive CRC patients undergoing curative surgery at our Institution were collected. Clinical data, tumour location, and AJCC staging of these patients are shown in Table 1. Primary tumour and matching normal tissue samples were obtained from a second cohort of 8 CRC patients, and used in a validation assay. Genomic DNA was isolated from peripheral blood samples using standard techniques. Tissue samples were immediately frozen in liquid nitrogen and stored at -80°C until nucleic acids extraction. The study was approved by the Ethics Committee at our Institution, and all patients gave their informed written consent.

**Table 1:** Clinical data of colorectal cancer (CRC) patients

	**CRC** n = 30 (%)
**Age (yrs):**	
Mean (± SD)	59 ± 14
**Sex:**	
Male/Female	15/15
**Tumour location:**	
Ascending colon	7 (23%)
Transverse colon	2 (7%)
Descending colon	4 (13%)
Sigmoid colon	8 (27%)
Rectum	4 (13%)
Rectum-Sigmoid colon	5 (17%)
**AJCC stage:**	
0	1 (3%)
I	3 (10%)
IIa	8 (27%)
IIIb	3 (10%)
IIIc	1 (3%)
IV	14 (47%)

**Age (yrs):**	
Mean age <50	41 ± 7
**CRC:**	
Familiar/Sporadic	1/8
**Tumour location:**	
Ascending colon	1 (11%)
Descending colon	2 (22%)
Sigmoid colon	4 (45%)
Rectum	1 (11%)
Rectum-Sigmoid colon	1 (11%)
**AJCC stage:**	
I	1 (11%)
IIIb	2 (22%)
IIIc	1 (11%)
IV	5 (55%)

**Age (yrs):**	
Mean age >50	66 ± 9
**CRC:**	
Familiar/Sporadic	8/13
**Tumour location:**	
Ascending colon	6 (29%)
Transverse colon	2 (9%)
Descending colon	2 (9%)
Sigmoid colon	4 (19%)
Rectum	3 (15%)
Rectum-Sigmoid colon	4 (19%)
**AJCC stage:**	
0	1 (5%)
I	2 (9%)
IIa	8 (38%)
IIIb	1 (5%)
IV	9 (43%)

### RNA extraction from fresh frozen tissue

About 150–200 mg fresh frozen tissues were used to isolate total RNA by phenol extraction (TRIzol Reagent, Invitrogen Corporation, Carlsbad, CA, USA) which was subsequently purified by column chromatography (RNeasy Mini Kit, Qiagen, Valencia, CA, USA). RNA integrity was monitored using MOPS gel electrophoresis.

### Cell culture, 5-Aza-CdR Treatments

The HCT116, CaCo2 and SW480 cell lines were purchased from the American Type Culture Collection (ATCC, Rockville, MD, USA), and maintained in DMEM medium (Invitrogen Corporation, Carlsbad, CA) supplemented with 10% FBS, 100 U/ml penicillin, and 100 μg/ml streptomycin in a humidified 5% CO_2 _atmosphere at 37°C. For demethylation studies, cells were seeded at a density of 1 × 10^6 ^cells per 100-mm dish, and incubated for 24 hrs in a growth media. Subsequently, 5-Aza-CdR (Merck Chemicals Ltd., Nottingham, UK) was added to the incubation mixture following two different protocols. In the acute treatment 1 μM 5-Aza-CdR was added to incubation mixture for 24 hrs; afterwards, the medium was changed once daily for 3 consecutive days; DNA and RNA content were checked at 2^nd^, 4^th ^and 6^th ^days [15,16]. In the chronic treatment, 2 μM 5-Aza-CdR was added for 24 hrs at day 1^st^, 3^rd ^and 5^th ^[17]; at each experimental day, the cells were placed in fresh medium and harvested at day 6^th ^to isolate DNA and RNA.

### qPCR Assay

For qPCR, 1.0 μg of total RNA from CRC cell lines and normal and tumour tissues of the second cohort of CRC samples was used with hexamer random primers to run the first strand cDNA synthesis by the RT-System kit (Promega Corporation, Madison, WI). Oligonucleotide sequences were designed by means of the PrimerExpress program (Applied Biosystems, Applera, Foster City, CA) with default parameters in every case; whenever possible, the oligos were designed to span an intron region (Table 2). To ensure specificity, amplicon sequences were checked by both BLAST and BLAT programs against the human genome. The efficiency of each oligo pairs was checked by diluting a series of control cDNAs. All qPCRs were performed in a 10-μl final volume, in three replicates per sample, set up in a 384-well plate format with the Biomek 2000 robot (Beckman Coulter, Inc., Miami, FL). The assays were run in an ABI 7900 Sequence Detection System (Applied Biosystems, Applera, Foster City, CA, USA) with the following amplification conditions: 50°C for 2 min, 95°C for 10 min, and 50 cycles at 95°C for 15 s and at 60°C for 1 min. Expression of mRNA from candidate genes was analysed quantitatively by means of SYBR Green Real Time PCR (Invitrogen Corporation, Carlsbad, CA) and raw Ct values calculated with SDS2.0.

**Table 2:** Primer Sequences and Conditions for qRT-PCR, Bisulfite sequencing and MSP Analysis

**Gene symbol**	**RefSeq mRNA**	**Primer Forward (5'-->3')**	**Primer Reverse (5'-->3')**	**Size (bp)**	**AT^† ^(°C)**
		***Quantitative Real Time PCR (qPCR) assay***			
*ABCA8*	NM_007168	CCATCATGGTATCTGGGAGGTT	GCAGGTAATCTTTGCCAAATTTG	77	60
*AQP8*	AB013456	CACGGGCTGGCTTTGG	CCAGTACGGGAGGAGCATCA	128	60
*CLCA4*	NM_012128	CAAAATGGCCTATCTCAGTATTCCA	TCGCTTTGGCTTGAAGATTGT	68	60
*HPGD1*	NM_000860	GCATGGCATAGTTGGATTCACA	AAGCCTGGACAAATGGCATT	83	60
*PRDX6*	NM_004905	GCCCTTTCAATAGACAGTGTTGAG	ATCGATGATGGGAAAAGGTAACTT	104	60
*SLC26A3*	NM_000111	ATCGTTGGAACTGATGATGACTTC	CAGCATCATGGATTGTTAAGAAAAA	90	60
*STX12*	NM_177424	AAGAAAGAGAAACGGCAATTCG	TCATGGCCAAATCTTTAAATATCTGA	75	60
*CSE1L*	NM_001316	CGGTTCAAACACAATAGCAAGTG	GGATGCAATCAGCTTCTGAAAGA	78	60
*HSPH1*	NM_00664	AACAAAATCCCAGATGCTGACA	ACCTTTATTTTGGGCTTTTTAGCTT	79	60
*NEBL1*	HSY1624	ATCCATGAGATCAATGCAGCAT	CATCCTGGGCACTGTAATCGT	73	60
*RFC3*	NM_002915	CTGAGGGAGACTGCAAATGCT	ACAGCCTTCCACGAACTTCAA	72	60
*SLC12A2*	NM_001046	AAGGAACATTCAAGCACAGCTAATATT	TGCCATGTAGAGAGCACTAGACACA	86	60
*SOX9*	NM_000346	ACGCCGAGCTCAGCAAGA	CACGAAGGGCCGCTTCT	70	60
*GTF2IRD1*	ENST00000265755	AATCTGCAATGATGCCAAGGT	CCGAGACCCGCTTTCTCTT	70	60
*MXI1*	NM_005962	CGGCACACAACACTTGGTTT	GGCTTTTTCTTTCAGCTTCTTCA	75	60
*NR3C2*	NM_000901	TTCATTCTCAGTACCAATAAAGCAAGA	GGTTTACTGTTGGATTCCCTTTAAAA	82	60
*SGK1*	NM_005627	AGGAGCCTGAGCTTATGAATGC	GACGACGGGCCAAGGTT	75	60
*NDRG2*	NM_201535	CTGACCGAGGCCTTCAAGTACT	GGCGAGTCATGCAGGATGA	66	60
*TPX2*	NM_012112	AGCCCTTTGTTCCCAAGAAAG	CCAGCTGAAAAGGTTCCTGAA	80	60
*UBE2C*	NM_181799	TGGAGCTTACTCTGCAACTGTTTC	CCAAATGCCAGAACCCAACATTGATAGTCC	74	60
*CCNB1*	NM_031966	CCTGGCTAAGAATGTAGTCATGGTAA	GCATGCTTCGATGTGGCATA	83	60
*SCNN1B*	ENST00000343070	CATTGAAGAATCAGCAGCCAATA	CCCATCCAGAAGCCAAACTG	74	60
*FOXM1*	U74613	AGCAAGCGAGTCCGCATT	CTGCAGAAGAAAGAGGAGCTATCC	68	60
*SGK2*	NM_016276	ACATCATTTACAGGGATCTGAAACC	TCCTTGCAGAGGCCAAAATC	87	60
					
		***Bisulfite Sequencing Analysis (BSA)***			
*NDRG2*		TTTTCGAGGGGTATAAGGAGAGTTTATTTT	CCAAAAACTCTAACTCCTAAATAAACA	320	53
*CSE1L*		GTTTGGAATTTTAGTATTTTGGGAG	CTCTAACCATACCAACAAACTTCAC	285	60
*HSP1*		GGAGAGGGTTTGGGTATGTAA	CAAAAAAATAAAATAAACCTAAAAAAC	194	56
*PRDX6*		TATTTTTTTGTAGGGAGTTGGT	TAACATCCTTCAAACACTATAAACC	279	56
*SOX9*		TTTTTATTGATTTTTTTTGTAAAAG	ATACCAAAATTTTAATACCTTCTCC	388	53
					
		***Methylation-Specific PCR (MSP) assay*^?^**			
*NDRG2_UnM*		AGAGGTATTAGGATTTTGGGTATGA	CCACTAAAAAAACAAAAATCTCACC	125	55
*NDRG2_M*		AGAGGTATTAGGATTTTGGGTACG	GCTAAAAAAACGAAAATCTCGC	123	55
*NDRG2_UnM_2*		GGTAAATTTATTTGGGTATTGA	CAAAAACAAAATTAACCCTACAAA	210	54
*NDRG2_M_2*		TAGTGGTAAATTTATTCGGGTATCG	CAAAAACGAAATTAACCCTACGA	214	62
*p16-UnM*		TTATTAGAGGGTGGGGTGGATTGT	CAACCCCAAACCCACAACCATAA	151	65
*p16-M*		TTATTAGAGGGTGGGGCGGATCGC	GACCCCCGAACCGCGAACCGTAA	150	68
*APC-UnM*		GTGTTTTATTGTGGAGTGTGGGTT	CCAATCAACAAACTCCCAACAA	108	63
*APC-M*		TATTGCGGAGTGCGGGTC	ACCACCTCATCATAACTACCCACA	98	63
*MLH1-UnM*		TTTTGATGTAGATGTTTTATTAGGTTGT	ACCACCTCATCATAACTACCCACA	124	60
*MLH1-M*		ACGTAGACGTTTTATTAGGGTCGC	CCTCATCGTAACTACCCGCG	115	60

?UnM = unmethylated sequence; M = methylated sequence.

† AT = annealing temperature.

### *In Silico *Search and Bisulfite Sequencing Analysis (BSA)

The presence of CpG islands, overlapping the 5'-UTR, was examined by means of the MethPrimer , according to CpG islands definition.

Bisulfite modification of DNA from colon cancer cell lines, peripheral blood and frozen tissues of patients was assayed, as reported by Herman *et al*. [18]; normal lymphocytes (NL) and *in vitro *methylated DNA (IVD) were used as negative and positive controls, respectively. In the assay, 1 μg of DNA was denaturated by treatment with NaOH at 37°C for 10 min, followed by incubation with hydroquinone and sodium bisulfite at 50°C for 16–17 h in the dark. After treatment, DNA was purified using DNA cleanup kit (Promega Corporation, Madison, WI), incubated with NaOH at 37°C for 15 min, precipitated with ammonium acetate and 100% ethanol, washed with 70% ethanol and, finally, re-suspended in 25 μl of distilled water. DNA methylation patterns in the CpG islands were determined by BSA using the primers listed in Table 2. The PCR conditions were 3 min at 94°C, 30 cycles of 94°C for 30 sec, specific annealing temperature for 30 sec, and 72°C for 60 sec. The sequence of the PCR products was analysed by using Sequencing Analysis 3.4.1 (Applied Biosystems, Applera, Foster City, CA, USA).

### MSP Assay

Qualitative analysis of CpG islands in the promoter region of the *NDRG2, p16, APC*, and *MLH1 *genes in 30 patients of CRC, in cell lines, in NL and IVD was carried out by MSP assay [18]. The primers for unmethylated and methylated DNA are listed in Table 2. For the *NDRG2 *promoter region we used two different primers (NDRG2_UnM/NDRG2_M and NDRG2_UnM2/NDRG2_M2) to cover the same region sequenced by the bisulfite assays. PCR reaction was carried out in a 25 μl mixture containing 0,2 mM each dNTP, 1.5 mM MgCl_2_, primers (10 μM each), bisulfite-modified DNA (50 ng), and 0.75 U of Amplitaq *Taq *Gold polymerase (Applied Biosystems, Applera, Foster City, CA) for 35 cycles (95°C for 12 min, 94°C for 1 min, TA for 1 min, then 72°C for 1 min, followed by a final extension at 72° for 5 min) and analysed on a 3% agarose gel stained with ethidium bromide. All reactions were run in duplicate to ensure consistent and reproducible results.

### Statistical Analysis

We carried out three separate statistical analyses. In the initial analysis, qPCR data were used to validate our previous array results [14]. Calculations were made using the Comparative CT method [19,20]. We used three genes, that is hEEF1A1, hGAPDH and hHRPT1, to normalize input cDNA for each sample, with cDNA of normal tissue used as calibrator. The chi-square method with one degree of freedom (χ^2^_1_), calculated by BMDP Statistical Software (BMDP Statistical Software, Cork Technology Park, Model Farm Road, Cork, Ireland) [21], was used to asses statistical significance of expression difference for each gene of the 8 paired samples. The second analysis concerned the variation of gene expression before and after 5'-Aza-CdR treatment by means of the T test, calculated by BMDP Statistical Software. Expression level of the post-treatment specimen compared to the pre-treatment specimen was calculated as a log-transformed ratio. A gene was classified as up-regulated following the 5-Aza-CdR treatment when relative mRNA expression was greater or equivalent to 1.65-fold in at least one treatment condition in one cell line. Genes with no change or very low expression levels in post treatment specimens were no further considered in the analysis.

The final part of our analysis evaluated the BSA data. The median number of full CpG islands, present in normal and tumour tissues, was calculated and compared in tumour (T) matched normal (N) tissue of the same patients. The χ^2^_1 _method was used to asses significant difference; a number of CpG islands higher than 5 was taken as statistically significant.

## Results

### qPCR validation of deregulated genes

Among the genes higlighted as significantly deregulated in our previous microarray study [14], 24 genes were selected for further validation by using the quantitative Real-Time PCR (qPCR), based on their cellular function, such as transport, signal transduction, intracellular and cell surface signalling, cell cycle, replication-repair of DNA, and protein folding. (Table 3).

**Table 3:** List of genes selected from microarrays analysis comparing normal mucosa matched tumour colon tissue. The different expression in tumoural tissue was showed.

**Function/category**	**Gene**	**Accession no**.	**Microarray Data**		**Gene description**
			**P value**	**Expression**	
Insulin Receptor Signaling	SGK1	NM_005627.1	0.059	down	Serum glucocorticoid regulated kinase (SGK)
Transport	CLCA4	NM_012128.2	0.052	down	chloride channel, calcium activated, family member 4
Transport	SLC26A3/DRA	NM_000111.1	0.044	down	solute carrier family 26, member 3 (SLC26A3)
Transport	AQP8	NM_001169.1	0.052	down	aquaporin 8
Transport	SCNN1B	NM_000336.1	0.046	down	sodium channel, nonvoltage-gated 1, beta (S. Liddle)
Transport (ATP binding)	ABCA8	NM_007168.1	0.040	down	ATP-binding cassette, sub-family A (ABC1), member 8
Protein transport	STX12	AI816243	0.051	down	syntaxin 12
Receptor activity (mineralcorticoid)	NR3C2	NM_000901.1	0.049	down	nuclear receptor subfamily 3, group C, member 2
Cell cycle (proliferation)	MXI1	NM_005962.1	0.053	down	MAX-interacting protein 1 (MXI1)
Cell cycle (differentiation)	NDRG2	NM_016250.1	0.039	down	N-myc downstream-regulated gene 2
Prostaglandin metabolism	HPGD	J05594.1	0.050	down	hydroxyprostaglandin dehydrogenase 15-(NAD)
Prostaglandin and leukotriene Metabolism	PRDX6	NM_004905.1	0.050	down	peroxidase, acidic calcium-independent phospholipase A2
Signal tansduction	SGK2	NM_016276.3	0.038	down	serumglucocorticoid regulated kinase (SGK)
Transcription factors	FOXM1	NM_021953.1	0.046	up	forkhead box M1 (FOXM1)
Transcription factors	GTF2IRD1	NM_016328.1	0.046	up	GTF2I repeat domain-containing 1 (GTF2IRD1)
Transcription factor	SOX9	NM_000346.1	0.045	up	Sex determining region Y-box 9
ATP-binding/proteins folding	HSPH1	BG403660	0.043	up	heat shock 105 kD (HSP105B)
Transport solute carrier	SLC12A2	NM_001046	0.048	up	solute carrier family 12
Cell cycle (proliferation)	TPX2	NM_012112.1	0.044	up	restricted expressed proliferation associated protein
Cell cycle (progression)	UBE2C	NM_007019.1	0.050	up	ubiquitin carrier protein E2-C (UBCH10)
Cell cycle (proliferation)	CSE1L/CAS	NM_001316	0.052	up	CSE1 chromosome segregation 1-like (yeast)
Signal Transduction	CCNB1	Hs.23960	0.048	up	cyclin B1
Focal adhesion	NEBL	NM_006393.1	0.038	up	nebulette protein (NEBL, actin-binding Z-disc protein)
Replication and repair	RFC3	BC000149	0.049	up	replication factor C (activator 1) 3 (38 kD)

qPCR assay was applied to analyse mRNA expression of the 24 selected genes, as well as of three control housekeeping genes (*GAPDH, EEF1A1 *and *HRPT1*) in the tumour and normal tissues taken from new cohort of 8 patients with CRC.

**Figure 1:**
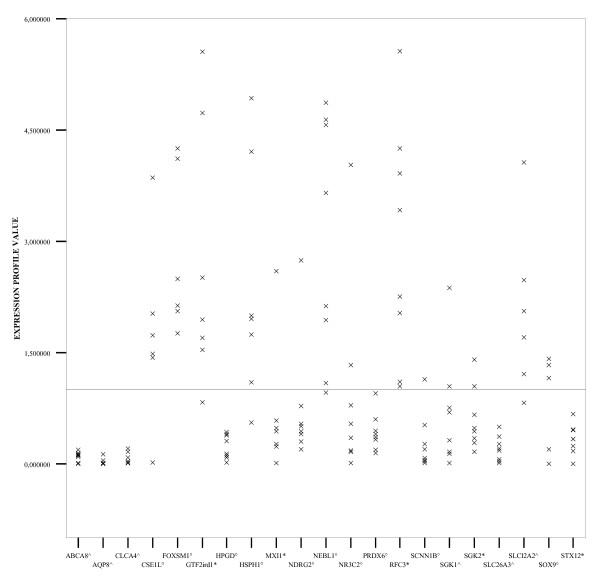
**Logarithmic expression profile value of twenty-one genes determined by quantitative reverse transcription-PCR by using the Comparative CT method**. Three housekeeping genes were used to normalize input cDNA for each sample with colorectal cancer, with cDNA of normal tissue used as calibrator. Crosses represents mean of triplicate determinations. *P < 0.05; ° P < 0.01; ^ P < 0.001.

Compared to normal tissue with an expression profile normalized to 1, in tumour samples 7 genes (*ABCA8, AQP8, CLCA4, HPGD1, PRDX6, SLC26A3*, and *STX12*) were uniformly under expressed in all 8 CRC patients, 3 genes (*MXI1, NDRG2 *and *SCNN1B*) in 7 patients, and 3 other genes (*SGK2, NR3C2*, and *SGK1*) in 6 patients. Eight genes, that is *CSE1L, GTF2IRD1, HSPH1, NEBL1, RFC3, SLC12A2, FOXM1 *and *SOX9*, were specifically over-expressed in tumour tissues from 7 patients (Fig. 1). The remaining 3 genes (*TPX2, UBE2C, CCNB1*) were excluded from the analysis because of indeterminate qPCR values. In general, qPCR results were in agreement with the microarray data.

### Gene expression before and after 5-Aza-CdR in colon cell lines

To investigate the role of methylated CpG islands in the modulation of gene expression, HCT-116, CaCo2 and SW480 human colon cancer cell lines were cultured with different doses of 5-Aza-CdR to induce a demethylation event, and gene expression levels were measured by means of qPCR. Two 5-Aza-CdR challenge regimens were used to obtain expression data under different cellular conditions: the acute treatment focused on moderate DNA demethylation to minimize cell viability, and the chronic treatment to maximize DNA demethylation (see Additional file 1).

**Figure 2:**
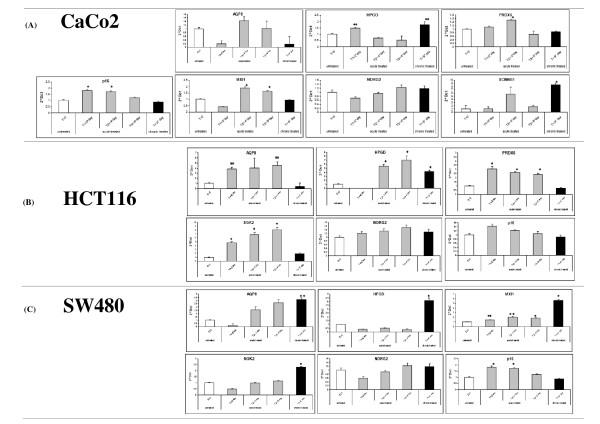
**Histograms depict expression levels of *AQP8, HPGD, PRDX6, MXI1, NDRG2, SCNNB1 *and *SGK2 *genes in CaCo2 cell line (A), HCT116 cell line (B) and SW480 cell line (C) before and after exposure to 5-Aza-CdR determined by quantitative real-time PCR using the Comparative CT method**. 2^^DCt ^indicates the ratio between the values of CT normalized to three housekeeping genes and compared to cDNA of untreated cells used as calibrator. Error bars indicate standard deviation from triplicate experiments. *p16 *gene was used as positive control in the experiments. Statistical significance threshold p-value (p < 0.001) for both acute and chronic treatment are shown. Asterisks indicate P < 0.05 values and double asterisks indicate P < 0.001 values.

Ten out of the 21 validated genes, i.e. *ABCA8, AQP8, HPGD, PRDX6, SLC26A3, STX12, NDRG2, MXI1, SGK2, and SCNNB1*, were analysed for the impact of their DNA hypermethylation on epigenetic events (Fig. 2). Other genes had no epigenetic influence and were, therefore, excluded from the analysis. Indeed, the underexpression of *NR3C2 *and *SGK1 *has been related to aldosterone regulation pathway [22], whereas the 14-3-3ε gene modulates the *CLCA4 *gene by interacting with the calmodulin-dependent pathway [23]. Demethylation of the 5'-UTRs of some genes with a concomitant increase in mRNA expression was documented in cell lines (Fig. 2). For *HPGD *and *NDRG2 *genes a 1.4 and 1.3-fold increase in CaCo2, a 7.0 and 1.6-fold increase in HCT116, and a 4.1 and 1.3-fold increase in SW480, respectively, was observed. PRDX6 gene expression increased 1.5 and 3.0-fold in Caco2 and HCT116 cells, respectively. MXI1 showed a 1.8 and 5.0-fold increase in Caco2 and SW480 cells, respectively (Fig. 2).

#### *In silico *search verification and Bisulfite Sequencing Analysis (BSA)

**Figure 3:**
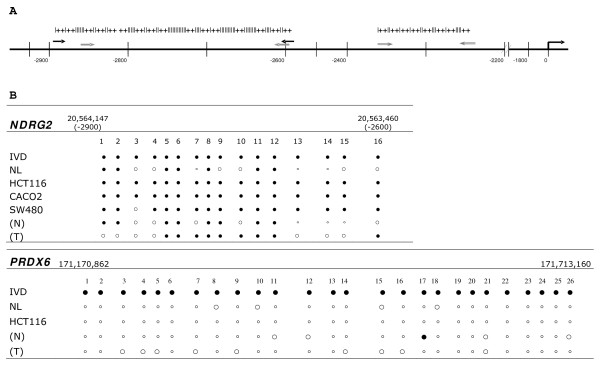
**CpG islands present in putative promoter regions of the two genes of interest**. A. Promoter structure of NDRG2 gene (the sequence are shown in Additional file 2). The black arrows correspond to NDRG2 primers for BSA assay, the grey arrows correspond to NDRG2_M_2 and NDRG2_M primers for MSP assays. B. CpG islands present in *NDRG2 *(numbered from 1 to 16) and *PRDX6 *(numbered from 1 to 26) genes obtained by MethPrimer software. Methylation status of CpG sites *in vitro* methylated DNA (IVD), normal lymphocytes (NL), three colon cancer cell lines, and in normal (N) and tumor (T) tissue of one patient with colorectal cancer. Methylated and unmethylated cytosine residues are indicated with *filled *and *small circles *while *open circles *denote partially methylated sites.

Two of the demethylated genes had CpG islands overlapping their putative promoter regions at *in silico *confirmation. By means of the MethPrimer software, we found 16 CpGs islands located between nucleotides 20,563,460 and 20,564,147 in *NDRG2 *genes (Fig. 3A and Additional file 2), and 26 CpGs islands located immediately at the 5' of the transcription start site and exon 1 (nucleotides 171,170,862 and 171,713,160) in the *PRDX6 *gene (Fig. 3A).

The methylation status of promoter regions of these putative tumour-suppressor genes was assessed by BSA in untreated cell lines, in tumour matched to normal tissues of one patient, *in vitro *methylated DNA (IVD) and in normal lymphocytes (NL). *PRDX6 *showed dense methylation only in IVD; *NDRG2 *showed a significant methylation in cell lines and in tumour tissue compared to normal tissue, suggesting a potential epigenetic regulation of the gene (Fig. 3B and Additional file 3). Full methylation in all 16 CpG sites of the *NDRG2 *gene was found in HCT116 and CaCo2 cell lines, and partial methylation at the 3^th ^CpG site in the SW480 cell line (Fig. 3B).

### Quantitation of NDRG2 methylation in paired tumour and normal tissue samples of CRC patients

**Figure 4:**
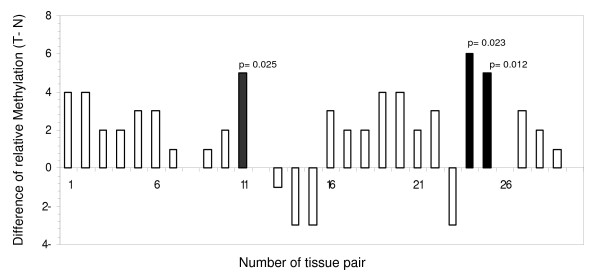
**Methylation analysis of the NDRG2 promoter in 30 tumour and normal tissue pairs, evaluated by the bisulfite sequencing analysis**. Data are expressed as the difference in methylation status between tumour and normal tissue.

To determine whether hypermethylation of the *NDRG2 *gene could be ascertained in primary CRC (Table 1), BSA of 30 primary colon tumour tissues matched to normal tissues was performed. When compared to their paired normal tissues, a relative increase of methylation in tumours was observed in 19 of 30 (63%) CRC patients (Fig. 4), but the increase was significant in only 3 patients (χ^2 ^> 5, df = 1, p < 0.05). In four tissue pairs, the relative methylation was apparently decreased, likely due a low sensitivity of the detection method. In the remaining patients figures were unchanged.

### Methylation-specific PCR (MSP) assay in colon cancer cell lines and primary CRC samples

MSP was performed to examine the methylation status of CpG islands identified in the *NDRG2 *gene (see Additional file 3). The methylation status of the gene was compared with that observed in three usually hypermethilated genes (*p16, APC*, and *MLH1) *in 3 colon cancer cell lines (HCT116, CaCo2 and SW480) and in 30 paired tumour-normal tissues. CpG methylation in *NDRG2 *was detected in all cell lines and in 8 of the 30 (27%) colorectal cancer patients (Table 4). No methylation was detected in 30 samples from normal tissue. Hypermethylation of *APC*, *p16*, and *MLH1 *genes in tumour tissue was found in 3 (10%), 4 (13%) and 6 (20%) patients, respectively, but not in normal colonic tissue (Table 5).

**Table 4:** Comparison of the clinicopathological features of 30 CRC patients according to the presence of NDRG2 methylation

	Number of CRC with *NDRG2 *methylation (%)	Number of CRC without *NDRG2 *methylation (%)	*p *value
**Age (yrs):**			
Age <50	4 (13.3)	5 (16.6)	ns
Age >50	4 (13.3)	17 (56.6)	
**Gender**			
Male	4 (26.7)	11 (73.3)	ns
Female	4 (26.7)	11 (73.3)	
**CRC:**			
Familiar	2 (25.0)	6 (75.0)	ns
Sporadic	6 (27.3)	16 (72.7)	
**Tumour location:**			
Proximal colon	2 (22.2)	7 (77.7)	ns
Distal colon	6 (28.6)	15 (71.4)	
			
**AJCC stage:**			
0	0	1 (4.8)	ns*
I	1 (12.5)	2 (9.5)	
IIa	1 (12.5)	7 (33.3)	
IIIb	0	3 (14.3)	
**IV**	**6 (75.0)**	**8 (38.1)**	**< 0,05^**

**MSI status^1^**			
High	1 (25.0)	3 (75.0)	ns
Low	1 (33.4)	2 (66.7)	
Stable	6 (27.3)	17 (72.7)	

Fisher exact test (2-tail); *Pearson Chi-square; ^Z test only for IV AJCC' stage

^1^Microsatellite instability (MSI) was determined by the mobility shift of PCR products using CC-MSI kit (AB Analitica s.r.l., Padova, Italy, ), that include the Bethesda panel microsatellite (BAT25, BAT26, D5S346, D17S250 and D2S123) and other four mononucleotide microsatellite loci (NR21, NR24, BAT40 and TGF*β*RII), in tumours. Tumours showing instability in four or more markers were classified as high MSI, those showing it in two marker as low MSI, and those showing no instability as microsatellite-stable.

**Table 5:** Promoter gene methylation rates in tumour and normal tissue from patients with colorectal cancer (CRC) sorted by tumour location

	**Proximal colon CRC (n = 9)**		**Distal colon CRC (n = 21)**	
	**Tumour**	**Normal**	**Tumour**	**Normal**
***APC***	0	0	3 (14%)	0
***p16***	1 (11%)	0	3 (14%)	0
***MLH1***	2 (22%)	0	4 (19%)	0

When relating the NDRG2 methylation status to clinical pathologic features, no association with age, gender, tumour site, and MSI status was observed. Conversely, a significant correlation was found between the *NDRG2 *methylation and the AJCC stage of the cancer (Z test, p < 0,05) (Table 4).

## Discussion

Carcinogenesis is a complex event characterized by the progressive development of genetic and epigenetic aberrations which ultimately result in loss of physiological control of cell growth and differentiation. The two most important epigenetic mechanism are represented by the DNA methylation, the conversion of cytosine into methyl-cytosine catalyzed by the DNA methyltransferase And histone modifications [24]. Changes in the DNA methylation pattern may occur everywhere in the DNA molecule. Global DNA hypomethylation generally occurs in centromeric repeats and repetitive sequences and contributes to carcinogenesis by causing chromosomal instability, reactivation of transposable elements, and loss of imprinting [25]. Hypermethylation is especially frequent in CpG islands, i.e. short DNA sequences rich in CpG dinucleotides, mostly located in the 5'-untranslated region (5'-UTR) of genes [24]. When CpG islands are heavily methylated, transcriptional gene silencing generally occurs. Although the fine mechanisms of regulation of the "epigenetic" machinery are still poorly understood, the DNA methylation may switch on or off several genes and, in particular, those regulating important biological phenomena, such as cell growth and differentiation [25]. In normal cells, this epigenetic mechanism is involved in several physiological events, such as the inactivation of X chromosome in female cells, silencing either paternal or maternal alleles of "imprinted" genes, and transcriptional blocking of exogenous integrated genes potentially dangerous for the cell life. However, aberrant DNA methylation is also relatively common in cancer cells and is likely to play an important role in cancer initiation and progression [26].

Since the pioneer studies of Baylin *et al*. [27], it has been widely recognized that cancer cells are characterised by two opposite events: a global hypomethylation which results in either up-regulation of proto-oncogenes and induction of genomic instability, favouring both uncontrolled cell growth [9] and mutations, and CpG islands hypermethylation of other genes, the so-called tumour-suppressor-genes (TSG), which contributes to loss of the negative control of the cell cycle [12]. Searching for up-regulated oncogenes and down-regulated TSG is important in basic science, especially when an epigenetic mechanism (hypomethylation or hypermethylation) is suspected. In fact, oncogenes and TSG not only may elucidate the highly complex molecular derangement in cancer cells, but also may be used as potential targets for new therapeutic approaches. DNA methylation is a reversible phenomenon which can be modulated by specific agents. An example is represented by demethylating drugs which can globally reduce the DNA methylation level of TSG promoters, restoring their normal activity. Interestingly, some *in vitro *experiments have shown that cancer cell lines reverted to normal phenotype after treatment with demethylating agent.

The current study was carried out with a three-step design. First, we specifically looked at up- and down-regulated genes not yet firmly associated with colon carcinogenesis, and selected 24 genes for validation with qPCR. A straight correlation between results obtained from qPCR and those from DNA microarray was found, implying that DNA microarray technology is a reliable tool to search for new genes significantly deregulated in cancer [28]. Second, we selected 10 of 21 genes (*ABCA8, AQP8, HPGD, PRDX6, SLC26A3, STX12, NDRG2, MXI1, SGK2, and SCNNB1*) as possible targets of epigenetic modifications in colon cancer, and after treatment with a demethylating agent, seven of them showed a significant increase of mRNA expression (AQP8, HPGD, PRDX6, MXI1, SCNNB1, SGK2 and NDRG2). From an *in silico *screening, only 2 genes (PRDX6 and NDRG2) were considered as possible candidates for the presence of CpG islands in their 5'-UTR. For the excluded genes, additional mechanisms of transcriptional regulation were hypothesized to be responsible for their differential expression. Third, to evaluate the methylation status of *PRDX6 *an *NDRG2 *genes in normal and cancer tissues, as well as in colon cancer cell lines, bisulphite sequencing analysis was used. In the PDRX6 gene the methylation status was not different from that observed in normal tissue. In the *NDRG2 *gene a significant methylation status either in colon cancer cell lines and in tumour tissue compared to normal tissue was observed. The underexpression of the *PRDX6 *protein responsible for the red-ox regulation of the cell, was found to be correlated with loss of function of *NKX3.1 *gene, known as TSG [29].

Using these approaches, the *NDRG2 *gene was selected for further analysis because: (i) it was suppressed in all colon cancer cell lines, (ii) its expression may be up-regulated in all cell lines by 5Aza-CdR treatments, and (iii) it is involved in important biological process such as cell growth [30], differentiation [31] and apoptosis [32]. The *NDRG2 *gene is a new member of the N-myc downstream-regulated gene (NDRG) family, that is located on chromosome 14q11.2 and encodes for a 41 kDa protein. It has been proposed that the NDRG2 gene is a candidate TSG, and its expression is low or undetectable in several primary tumour and tumour cell lines [30,33,34]. Liu *et al*. [35] revealed that the down-regulation reported in cancer be driven by promoter methylation, mutation, and genomic deletion of the *NDRG2 *gene. Recently, it has been shown that expression of the NDRG2 protein is modulated by the insulin-stimulated Akt-dependent phosphorylation [36]. Several studies have suggested that the NDRG2 mRNA is down-regulated or undetectable in a number of human primary cancers, such as squamous cell carcinoma, pancreatic cancer [37], glioblastoma [30], and cancer cell-lines. Recently, Zhang *et al*. [38] have demonstrated that c-Myc represses *NDRG2 *gene expression via Miz-1-dependent interaction with NDRG2 core promoter region, and this inverse regulatory relationship induces cell differentiation and proliferation.

The MSP assay was used to check for *NDRG2 *methylation status in 30 primary colon tumour tissues compared to normal colonic mucosal samples. After sorting colon cancer patients by age, gender, tumour site, and MSI status, no statistically significant association was observed between these features and the NDRG2 methylation. Nevertheless, there was a trend towards NDRG2 methylation status with an advanced tumour stage of the CRC samples, with significant value detected in patients with AJCC stage IV (p < 0.05). These results are in agreement with those reported in other cancer types [34,39] where *NDRG2 *expression is reduced in high-grade compared to low-grade tumours. In particular, Lorentzen *et al*. [39] suggested that in CRC samples the down-regulation of *NDRG2 *expression occurs during the progression from adenoma to carcinoma.

## Conclusion

In conclusion, we showed that NDRG2 expression is frequently suppressed in colon cancer cell lines in conjunction with aberrant DNA methylation, and that the loss of expression of this gene could be related to advanced colon tumour stage.

## Competing interests

The authors declare that they have no competing interests.

## Authors' contributions

AP, FP and AA wrote the manuscript with edits from all co-authors. RC designed and performed the methylation experiment. GM and BA designed and performed the qPCR experiment. MC designed the microarray patterns. RM, AD, NA performed the statistical analysis of microarray. SF performed the statistically analysis. MC deposited the data in Array Express. AP and FP conceived the project. All authors have read and approved the manuscript.

The microarray data are accessible through ArrayExpress accession number E-MTAB-57.

## Pre-publication history

The pre-publication history for this paper can be accessed here:



## Supplementary Material

Additional File 1 Supplementary Table. In table are shown the expression value (2^DCt), error standard (SE), t-test and p-value of *ABCA8, AQP8, HPGD, PRDX6, SLC26A3, STX12, ENACB1, SGK2, MXI1, NDRG2 *and *p16 *genes determined by quantitative real-time PCR, using the Comparative CT, before and after exposure to 5-Aza-CdR. 2^DCt indicates the ratio between the values of CT normalized to three housekeeping genes and compared to cDNA of untreated cells used as calibrator. Two 5-Aza-CdR challenge regimens (acute and chronic treatment) were used to obtain expression data. Click here for file

Additional File 2 Sequence of *NDRG2 *promoter gene. Sequence of *NDR2 *gene promoter region located between 20,564,147 and 20,563,460 nucleotides. The 300-bp region contains 16 CpG (boxes, numbers are indicated above) was analyzed by both bisulfite-sequencing and methylation specific PCR and the position of the primers (see Table 2) are indicated by horizontal black and grey arrows, respectively. Primers NDRG2_M_2 (grey arrows) was downstream and amplify a fragment of 124-bp. Click here for file

Additional File 3 Bisulfite-sequencing assay (BSA) and Methylation-specific PCR (MSP). **A) **Demostration of NDRG2 promoter methylation by bisulfite-sequencing from: *in vitro *methylated DNA (IVD), normal lymphocytes (NL), CaCo2 cell line, normal (N) and tumour (T) tissue of one patient. Note methylation of 4 depict CpG islands (CpG sites 13–16). **B) **Methylation-specific PCR of NDRG2 gene in two colon cancer cell lines (HCT116 and CaCo2), in normal lymphocyte (NL) and *in vitro *methylated DNA (IVD). ***U***, primers specific for unmethylated DNA; ***M***, primers specific for methylated DNA. Click here for file
