# Supplementary material for: Promoter methylation correlates with reduced NDRG2 expression in advanced colon tumour
Source: BMC Med Genomics. 2009 Mar 3;2:11. doi: 10.1186/1755-8794-2-11 (PMC2660908; doi:10.1186/1755-8794-2-11)
Supplement: Additional File 1 — Supplementary Table. In table are shown the expression value (2^DCt), error standard (SE), t-test and p-value of ABCA8, AQP8, HPGD, PRDX6, SLC26A3, STX12, ENACB1, SGK2, MXI1, NDRG2 and p16 genes determined by quantitative real-time PCR, using the Comparative CT, before and after exposure to 5-Aza-CdR. 2^DCt indicates the ratio between the values of CT normalized to three housekeeping genes and compared to cDNA of untreated cells used as calibrator. Two 5-Aza-CdR challenge regimens (acute and chronic treatment) were used to obtain expression data. [file 1755-8794-2-11-S1.pdf]

Caco2 Cell line																		
	untreated		acute treated												chronic treated			
	**T=0**		**T1=2°day**				**T2= 4°day**				**T3= 6°day**				**T= 6° day**			
**gene**	**N***	**SE**	**2^ΔCt**	**SE**	**t-test**	**p**	**2^ΔCt**	**SE**	**t-test**	**p**	**2^ΔCt**	**SE**	**t-test**	**p**	**2^ΔCt**	**SE**	**t-test**	**p**
ABCA8	1	0.035387	0.089114433	0.02147634	5.72	0.00462	NE	NE	NE	NE	NE	NE	NE	NE	5.13E-05	1.44E-05	32.10	0.00001
AQP8	1	0.093355	0.187501424	0.158319327	3.65	0.02183	1.449506	0.245559	1.45	0.22069	1.010323	0.409064	1.45	0.22061	0.169572	0.398353	1.79	0.14722
HPGD	1	0.041863	1.478376265	0.048846732	5.49	**0.00537**	0.701156	0.041861	3.73	0.02036	0.529786	0.336393	2.46	0.06952	1.736625	0.230069	2.74	**0.05167**
PRDX6	1	0.019739	1.12421931	0.046659115	2.18	0.09482	1.521059	0.047348	9.03	**0.00083**	0.716116	0.161489	1.96	0.12147	0.835188	0.049893	2.74	0.05211
SLC26A3	1	0.088622	0.327545788	0.12135806	3.23	0.03206	0.710518	0.037407	2.33	0.08055	NE	NE	NE	NE	3.863546	0.509609	4.80	**0.00865**
STX12	1	0.080558	0.369223875	0.022157253	6.25	0.00334	0.663835	0.038555	2.86	0.04570	NE	NE	NE	NE	0.400579	0.080118	3.77	0.01957
ENACB1	1	1.042202	1.084513898	0.560334449	0.05	0.96047	5.656337	2.161523	1.45	0.21977	1.688434	0.448911	0.60	0.58182	8.506657	0.584893	4.61	**0.00993**
SGK2	1	0.062545	0.397894526	0.045547526	5.57	0.00509	0.841086	0.011385	2.15	0.09804	0.80475	0.018042	6.40	0.00306	0.328767	0.046594	6.15	0.00355
MXI1	1	0.06342	0.415579014	0.012026856	7.75	0.00150	1.877348	0.124123	4.68	**0.00946**	1.628185	0.061483	16.50	**0.00008**	0.927679	0.040018	0.70	0.52297
NDRG2	1	0.112166	0.69813754	0.076115955	1.60	0.18414	0.920031	0.077069	0.42	0.69432	1.229442	0.138752	2.47	0.06875	1.177316	0.148233	0.68	0.53329
p16	1	0.066016	1.808466902	0.098934248	4.90	**0.00804**	1.72062	0.055768	5.92	**0.00409**	1.219486	0.045383	4.08	0.01508	0.874914	0.060754	0.99	0.37964

HCT116 Cell line																		
	untreated		acute treated												chronic treated			
	T=0		T1=2°day				T2= 4°day				T3= 6°day				T= 6° day			
**gene**	**N***	**SE**	**2^ΔCt**	**SE**	**t-test**	**p**	**2^ΔCt**	**SE**	**t-test**	**p**	**2^ΔCt**	**SE**	**t-test**	**p**	**2^ΔCt**	**SE**	**t-test**	**p**
ABCA8	1	0.424396	2.07754E-05	2.48014E-05	2.36	0.07799	0.000675	0.001362	2.35	0.07876	0.000244	0.000152	2.35	0.07810	0.000672	0.001088	2.35	0.07863
AQP8	1	0.14967	3.868762279	0.281421249	3.40	**0.02736**	4.043341	1.912731	1.23	0.28636	4.492586	0.729985	2.70	**0.05404**	0.388681	0.699448	0.48	0.65357
HPGD	1	0.140897	0.010029721	0.004444465	6.81	0.00243	5.490721	0.530414	6.69	**0.00260**	7.026243	1.026742	5.16	**0.00669**	4.172525	0.311997	7.01	**0.00219**
PRDX6	1	0.044341	3.087945414	0.264428	6.76	**0.00249**	2.612438	0.13046	9.22	**0.00077**	2.37328	0.187801	5.92	**0.00409**	0.751392	0.033232	3.20	0.03275
SLC26A3	NE	NE	NE	NE	NE	NE	NE	NE	NE	NE	NE	NE	NE	NE	NE	NE	NE	NE
STX12	1	0.068586	0.635829746	0.104740199	2.10	0.10353	2.234595	0.346035	2.98	0.04083	0.631858	0.086725	2.37	0.07679	1.288183	0.14717	1.34	0.25258
ENACB1	1	0.174676	0.195761505	0.026923313	3.99	0.01627	0.252303	0.030677	3.64	0.02194	0.122541	0.013965	4.65	0.00965	0.120611	0.022885	4.45	0.01124
SGK2	1	0.080778	4.785189582	0.215353064	12.78	**0.00022**	6.817452	0.510919	9.83	**0.00060**	8.103893	0.479917	12.67	**0.00022**	1.86905	0.320032	2.17	**0.09600**
MXI1	1	0.050839	1.102598721	0.035843689	1.18	0.30210	0.910596	0.050764	0.88	0.42858	1.079321	0.111258	0.49	0.65022	0.719068	0.06314	2.46	0.06934
NDRG2	1	0.090348	1.222303172	0.097428925	1.18	0.30201	0.83548	0.03671	1.29	0.26506	1.325456	0.130911	1.47	0.21527	1.179835	0.149252	0.75	0.49465
p16	1	0.056657	1.418138234	0.103596902	2.61	0.05947	1.215826	0.034595	2.37	0.07723	1.058408	0.116289	0.34	0.75255	0.894012	0.095301	0.70	0.52392

	untreated		acute treated												chronic treated			
**SW480 Cell line**	**T=0**		**T1=2°day**				**T2= 4°day**				**T3= 6°day**				**T= 6° day**			
**gene**	**N***	**SE**	**2^ΔCt**	**SE**	**t-test**	**p**	**2^ΔCt**	**SE**	**t-test**	**p**	**2^ΔCt**	**SE**	**t-test**	**p**	**2^ΔCt**	**SE**	**t-test**	**p**
ABCA8	1	0.912217	0.712563093	0.502388745	0.20	0.84890	0.568347	0.751227	0.26	0.80806	NE	NE	NE	NE	0.000672	0.001088	1.09	0.33534
AQP8	1	0.054127	0.17487	0.27008	1.01	0.37146	2.58068	0.47821	1.54	0.19916	3.68423	0.52447	2.50	0.06696	4.14080	0.30701	3.66	**0.02152**
HPGD	1	0.015041	0.360332121	0.021355609	17.58	0.00006	0.504238	0.02155	13.55	0.00017	0.335066	0.094254	6.08	0.00369	4.172525	0.311997	9.70	**0.00063**
PRDX6	1	0.042575	0.776651169	0.054516093	2.30	0.08290	0.784952	0.019654	3.46	0.02592	0.577111	0.261629	1.39	0.23684	0.751392	0.033232	3.28	0.03052
SLC26A3	NE	NE	NE	NE	NE	NE	NE	NE	NE	NE	NE	NE	NE	NE	NE	NE	NE	NE
STX12	1	0.064585	0.267196435	0.02028343	8.63	0.00099	0.537044	0.045033	4.22	0.01344	0.217257	0.224323	2.71	0.05358	1.288183	0.14717	1.36	0.24517
ENACB1	1	0.124284	0.05960	0.00521	7.26	0.00191	0.17193	0.02092	5.70	0.00467	0.19152	0.01953	5.62	0.00492	0.31419	0.04398	4.08	0.01515
SGK2	1	0.031906	0.48652	0.05598	5.84	0.00428	0.97256	0.08170	0.24	0.82101	1.14998	0.07243	1.44	0.22394	2.28295	0.05229	15.24	**0.00011**
MXI1	1	0.047886	1.41140	0.08521	3.09	**0.03654**	2.04333	0.18010	4.58	**0.01021**	1.81148	0.08103	6.29	**0.00326**	5.66167	0.13164	25.97	**0.00001**
NDRG2	1	0.112166	0.59813754	0.076115955	2.13	0.09972	0.915871	0.077069	0.44	0.67962	1.225876	0.138752	0.90	0.41891	1.177229	0.148233	0.68	0.53348
p16	1	0.066016	1.808466902	0.098934248	4.90	**0.00804**	1.72062	0.055768	5.92	**0.00409**	1.219486	0.045383	1.97	0.12013	0.874914	0.060754	0.99	0.37964

N: normalized value*

NE indicates that the gene is not expressed in this cell line.
