# Supplementary figures and images for: Promoter methylation correlates with reduced NDRG2 expression in advanced colon tumour
Source: BMC Med Genomics. 2009 Mar 3;2:11. doi: 10.1186/1755-8794-2-11 (PMC2660908; doi:10.1186/1755-8794-2-11)

Additional file 2

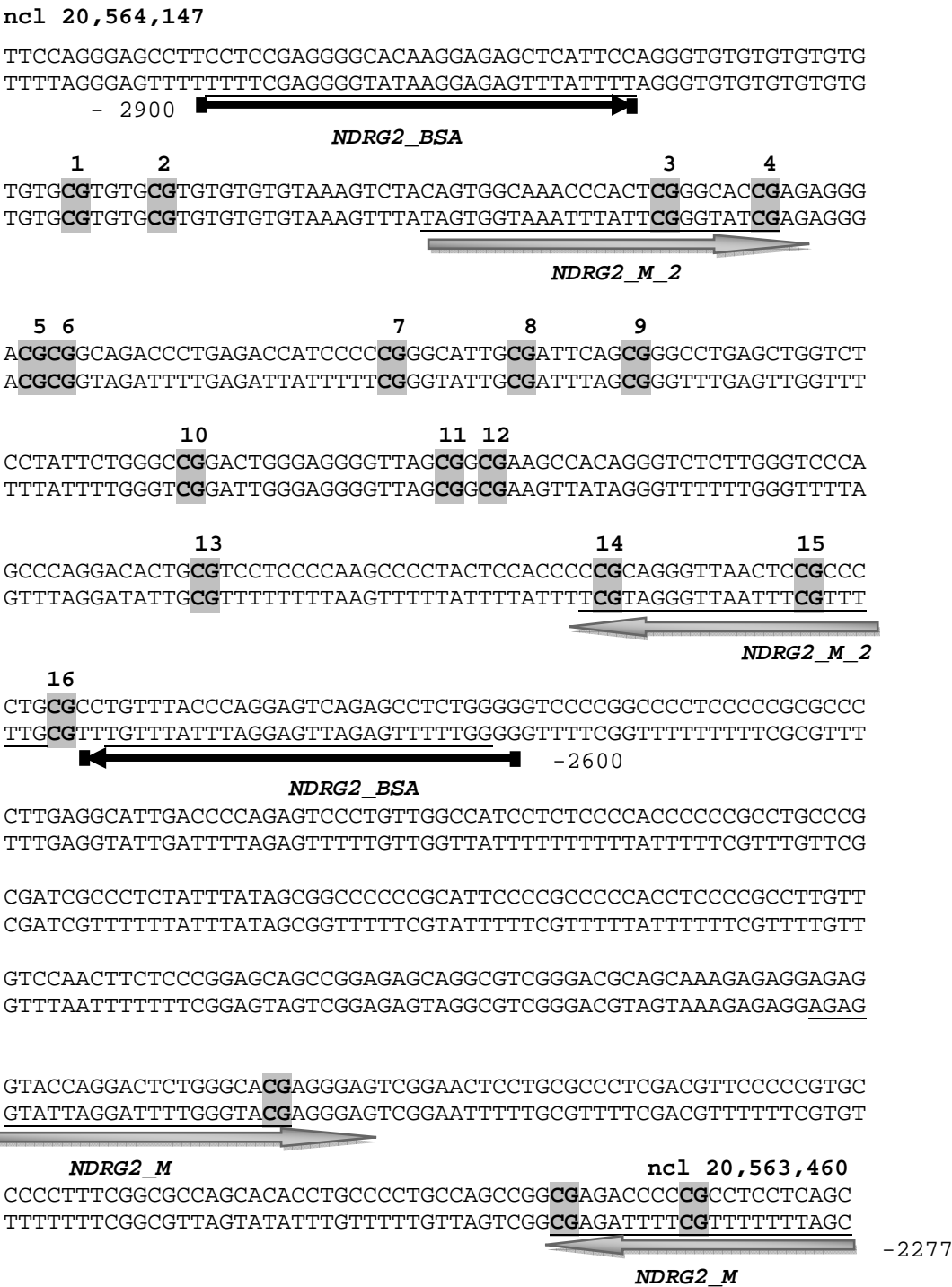

Supplement: Additional File 2 — Sequence of NDRG2 promoter gene. Sequence of NDR2 gene promoter region located between 20,564,147 and 20,563,460 nucleotides. The 300-bp region contains 16 CpG (boxes, numbers are indicated above) was analyzed by both bisulfite-sequencing and methylation specific PCR and the position of the primers (see Table 2) are indicated by horizontal black and grey arrows, respectively. Primers NDRG2_M_2 (grey arrows) was downstream and amplify a fragment of 124-bp. [file 1755-8794-2-11-S2.pdf]

**A**

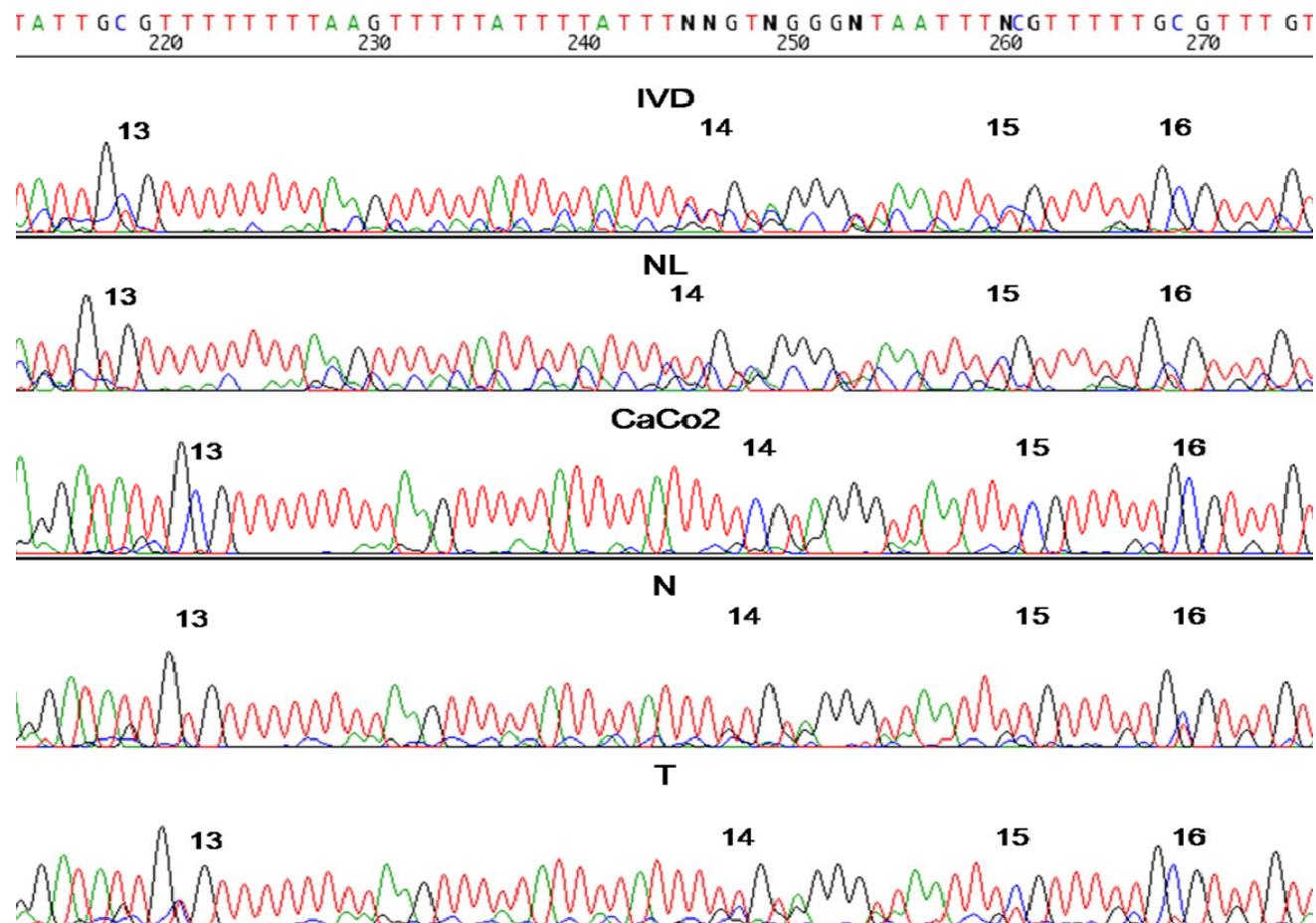

**B**

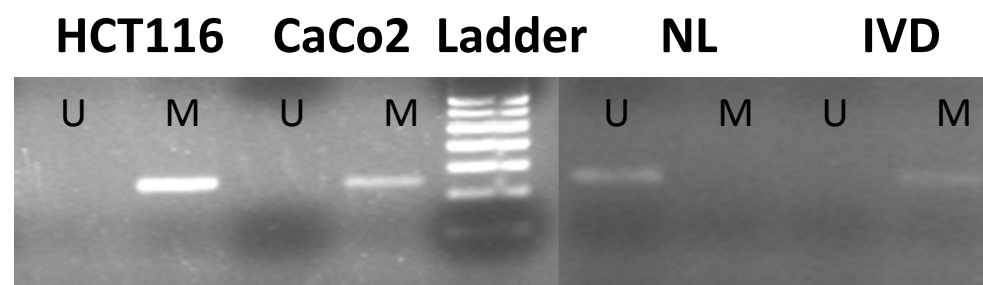

Supplement: Additional File 3 — Bisulfite-sequencing assay (BSA) and Methylation-specific PCR (MSP). A) Demostration of NDRG2 promoter methylation by bisulfite-sequencing from: in vitro methylated DNA (IVD), normal lymphocytes (NL), CaCo2 cell line, normal (N) and tumour (T) tissue of one patient. Note methylation of 4 depict CpG islands (CpG sites 13–16). B) Methylation-specific PCR of NDRG2 gene in two colon cancer cell lines (HCT116 and CaCo2), in normal lymphocyte (NL) and in vitro methylated DNA (IVD). U, primers specific for unmethylated DNA; M, primers specific for methylated DNA. [file 1755-8794-2-11-S3.pdf]
